# Evaluation of the effect of agroclimatic variables on the probability and timing of olive fruit fly attack

**DOI:** 10.3389/fpls.2024.1401669

**Published:** 2024-07-15

**Authors:** Gabriele Rondoni, Elisabetta Mattioli, Vito Antonio Giannuzzi, Elena Chierici, Andrea Betti, Gaetano Natale, Ruggero Petacchi, Franco Famiani, Antonio Natale, Eric Conti

**Affiliations:** ^1^ Department of Agricultural, Food and Environmental Sciences, University of Perugia, Perugia, Italy; ^2^ TeamDev – Software, GIS and Web Engineering, Perugia, Italy; ^3^ O.P.O.O., Perugia, Italy; ^4^ Center of Plant Sciences, Scuola Superiore Sant’Anna, Pisa, Italy

**Keywords:** *Bactrocera oleae*, Diptera, insect monitoring, Oleaceae, oviposition, pest management, Tephritidae

## Abstract

Agroclimatic variables may affect insect and plant phenology, with unpredictable effects on pest populations and crop losses. *Bactrocera oleae* Rossi (Diptera: Tephritidae) is a specific pest of *Olea europaea* plants that can cause annual economic losses of more than one billion US dollars in the Mediterranean region. In this study, we aimed at understanding the effect of olive tree phenology and other agroclimatic variables on *B. oleae* infestation dynamics in the Umbria region (Central Italy). Analyses were carried out on *B. oleae* infestation data collected in 79 olive groves during a 7-year period (from 2015 to 2021). In July–August, *B. oleae* infestation (1% attack) was negatively affected by altitude and spring mean daily temperatures and positively by higher winter mean daily temperatures and olive tree cumulative degree days. In September–October, infestation was negatively affected by a positive soil water balance and high spring temperatures. High altitude and cumulative plant degree days were related to delayed attacks. In contrast, high winter and spring temperatures accelerated them. Our results could be helpful for the development of predictive models and for increasing the reliability of decision support systems currently used in olive orchards.

## Introduction

*Olea europaea* L. is one of the oldest and most abundant tree crops in the Mediterranean regions, where it is of essential socioeconomic and ecological importance (Michalopoulos et al., 2020; Caselli and Petacchi, 2021). Approximately 95% of the global demand for olive oil is satisfied by southern European countries, with Spain, Italy, and Greece being the main olive oil producers (Fraga et al., 2021). Given the importance of olive cultivation, it is imperative to effectively manage pests that can cause significant production losses. More than 255 species, including insect pests, mites, nematodes, and pathogenic microorganisms, are potentially harmful to *O. europaea* (Caselli and Petacchi, 2021). Most of the yield loss is caused by the key pest *Bactrocera oleae* (Rossi) (Diptera: Tephritidae). In addition, moths [e.g., *Prays oleae* (Bernard)] contribute locally or occasionally to yield decline (Caselli and Petacchi, 2021). Insects can also be pathogen vectors, such as *Philaenus spumarius* L., the primary vector of *Xylella fastidiosa* subsp. *pauca*, which is responsible for the olive quick decline syndrome (Elbeaino et al., 2014; Sevarika et al., 2022). In a scenario of climate change, the ability to predict the evolution of the cycle of pests and plants, assessing the risks associated with their interaction, represents a critical challenge for the implementation of a proper control strategy.

Herbivorous insects, especially those with low thermal thresholds, are highly sensitive to changes in climate (Deutsch et al., 2008). Climate change may alter plant–pest phenological events, such as flowering and leaf unfolding, insect overwintering, and migration (Gordo and Sanz, 2005). Depending on the insect species, higher mean daily temperatures and extreme climate events, no longer sporadic, could cause the extension of suitable geographical ranges for herbivores but also the disruption of the synchronization of biological cycles among herbivores and their natural enemies (Forrest, 2016; Bonsignore et al., 2020; Skendžić et al., 2021), with an expected substantial increase in crop losses (Deutsch et al., 2018). As a result, monitoring methods and pest management programs must be reviewed and, if necessary, adapted to the new climatic changes that are occurring. In recent decades, pest control relied mainly on the use of broad-spectrum insecticides, with negative effects related to the decline of natural enemies, the occurrence of insecticide resistance in the target population, environmental pollution, and human health (Damos et al., 2015). Integrated pest management (IPM) is now commonly adopted, foreseeing the combined use of synthetic insecticides with more sustainable methods (Stetter and Lieb, 2000). The use of successful prediction models can play a pivotal role within IPM, also in combination with machine learning algorithms, used to manage complex datasets (McQueen et al., 1995; Damos, 2015; Raza et al., 2015; Benos et al., 2021; Caselli and Petacchi, 2021; Skendžić et al., 2021). Degree day models are a conventional instrument for predicting insect phenology (AliNiazee, 1979; Jones et al., 1991; Song et al., 2003; Rebaudo and Rabhi, 2018; Barker et al., 2020). However, physiologically based population modeling merges information on insect development and crop phenology to achieve more accurate predictions (Gutierrez et al., 2009; Rossini et al., 2022). Since a precise indication of pest outbreaks is necessary in an IPM context, machine learning has been applied in decision support systems (Capalbo et al., 2017; Ip et al., 2018; Rossini et al., 2022). A decision support system is a set of computer programs, mathematical models, and heuristic information that operate synergistically to improve decision-making (Nestel et al., 2019).

The olive fruit fly, *B. oleae* is an important pest of *Olea* spp. in Europe, Asia, Africa, and North America (Varikou, 2022). *Bactrocera oleae* is expected to expand due to global warming, thus colonizing areas at higher latitudes and altitudes (Petacchi et al., 2015; Marchi et al., 2016). Furthermore, an increase in average temperatures can affect the adult phenology in spring or can halt egg development in summer, but, on the other side, it can prolong the oviposition period in autumn, possibly resulting in increased yield losses (Caselli and Petacchi, 2021). The economic damage of *B. oleae* is estimated at more than one billion US dollars per year, only in the Mediterranean region (Van Asch et al., 2015).

The larval stage is responsible for both qualitative and quantitative damage due to its feeding activity within the olive mesocarp, leading to a strong decline in oil quality and premature fruit drop (Gömez-Caravaca et al., 2008). IPM programs against *B. oleae* are primarily based on monitoring of adults, sampling of olives to evaluate active infestation, and eventually pesticide treatments. In recent years, understanding the population dynamics of *B. oleae* has become a major focus of research on this pest. Since dimethoate use has been banned due to its toxic effects (Commission Implementing Regulation (EU), 2019/1090), alternative prevention-based control strategies are now recommended. Olive orchard monitoring is required due to the numerous factors that affect *B. oleae* infestations such as temperature, weather conditions, geographical location, olive tree variety, and management practices (Wang et al., 2009, 2013; Johnson et al., 2011; Rizzo et al., 2012; Petacchi et al., 2015; Volpi et al., 2020). Moreover, the olive variety can determine the fruit susceptibility to *B. oleae* attacks. Preferences are based on factors such as the size and shape of the fruits and their concentration of phenolic compounds (Varikou et al., 2022; González-Fernández et al., 2023). Furthermore, the mineral element content of the fruit may also influence female choice, making fruits that contain higher amounts of K and Fe more susceptible to attacks (Garantonakis et al., 2016). Concerning the use of digital tools, so far, some predictive models, machine learning algorithms, and decision support systems have been used for *B. oleae* monitoring and control (Ordano et al., 2015; Petacchi et al., 2015; Marchi et al., 2016; Miranda et al., 2019; Benhadi-Marín et al., 2020; Volpi et al., 2020; González-Fernández et al., 2023). However, proper calibration of these tools for the Umbria region (Central Italy) is lacking. To fill the knowledge gap about the effect of climate and environment on *B. oleae* in the Umbria region, we analyzed the dynamics of *B. oleae* infestation over 7 years (from 2015 to 2021) in 79 olive groves in total.

## Material and methods

### Bactrocera oleae infestation data

Analyses were conducted on *B. oleae* infestation data with the collaboration of the Umbrian Olive Oil Producer Association (O.P.O.O.) operating in the Umbria region (Central Italy). The dataset accessed contains monitoring data collected from 2015 to 2021 by expert field technicians. Surveys included a total of 79 olive orchards. Fruits were sampled weekly from the second half of July (pit hardening) until harvest. Each sample consisted of 100 olives randomly collected from different plants (one fruit per plant) (Quaglia et al., 1982). The olives were visually inspected in the laboratory with a stereomicroscope. Healthy olives were separated from those with oviposition punctures (Daher et al., 2022). Fruits exhibiting oviposition punctures were dissected with a scalpel and observed under a stereomicroscope to assess the presence of *B. oleae*. Alive eggs and larvae were considered for calculating the active infestation index (calculated as in Delrio and Prota, 1977 and Tsolakis et al., 2011).

### Variables associated with *Bactrocera oleae* infestation

Several environmental, morphometric, and weather variables have been correlated, over time, to the olive fruit fly infestations, such as precipitations, altitude, elevation, and distance from the water (Torres-Villa et al., 2006; Volpi et al., 2020). Based on these studies, we identified a set of candidate explanatory variables related to weather (average daily air temperature, daily precipitation), morphometric (altitude, slope, exposition), and environmental (distance from lakes) parameters. Weather data used were obtained from the regional monitoring network—Regional Hydrographic Service (https://annali.regione.umbria.it/#). The monitoring network consists of sensors that send data in real time to the central station through radio links distributed throughout the territory, which manages the peripherals and stores the data. Daily observed data on air temperature and precipitation from 116 meteorological stations for the period 2014–2021 have been accessed and processed. Data processing included mapping of weather stations, time series charting and analysis, evaluation and removal of records with missing values, and evaluation and removal of records with outlier values. Weather data were georeferenced on the respective sensors’ positions. The point data were then used to interpolate and create weather surfaces. To create a surface of predicted temperature values for the region using the sample data, the Geostatistical Wizard in ArcGIS Pro was used (Apaydin et al., 2004; Kim et al., 2010; Merbitz et al., 2012; Antal et al., 2021; Katipoğlu, 2022). Geostatistical techniques quantify the spatial autocorrelation among measured points and account for the spatial configuration of the sample points around the prediction location. The inverse distance weighted (IDW) method was used to create the daily interpolated surfaces for the weather parameters over the 7-year period. The meteorological surfaces produced by the model were used to calculate the daily maximum, minimum, and average air temperature (°C) and daily precipitation (mm) for each monitoring record at its specific position. Those parameters relevant to model selection are reported in **Table 1**.

**Table 1:** List of all variables calculated and evaluated in the final models.

Variable	Description	Unit
**TEMP (NOV–FEB)**	Average daily temperatures during the November–February period	°C
**TEMP (MAR–MAY)**	Average daily temperatures during the March–May period	°C
**TEMP (−7D)**	Average of mean air temperatures in the 7 days prior to the monitoring day	°C
**SWB**	Soil water balance in the 30 days prior to the monitoring day	mm
**CDD (PLANT)**	Cumulative degree day with a lower threshold of 5°C	
**DEM**	Altitude (above the sea level)	m
**LAKES**	Euclidean distance from lakes	m

#### Calculation of agrometeorological variables

Weather data and weather surfaces have been used to calculate a set of variables, aiming at the identification and description of climatic drivers influencing *B. oleae* infestation. The agrometeorological variables were selected according to the annual cycle of *B. oleae* (Koveos, 2001) and based on available methodology (Volpi et al., 2020). The bioclimatic variables identified (**Table 1**) refer to three periods: 1) winter period, calculated for each year in the period November (of the previous year)–February; 2) spring period, calculated for each year in the period March–May; and 3) summer period, calculated from the beginning of June to the day of the attack, or calculated in the 7 days prior to the day of the attack. Another variable was chosen to consider the soil water balance and, therefore, refers to the water status of the olive grove, calculated in the 30 days before the day of the attack. The soil water balance was calculated as proposed by Hargreaves and Samani (1985) and adopted in Volpi et al. (2020). The cumulative degree days were calculated using the package “TrenchR” in the R environment (R Core Team, 2022; Buckley et al., 2023). Olive tree phenology was considered by calculating the cumulative degree day from January, with a lower threshold of 5°C [CDD (PLANT)] (Volpi et al., 2020). Other variables were originally calculated and considered, the cumulative degree day with a lower threshold of 8.99°C and an upper threshold of 30°C [CDD (INSECT)] (according to thresholds in Crovetti et al., 1982; Gonçalves and Torres, 2011), or the cumulative precipitation (in mm) during summer (RAIN) (Volpi et al., 2020), but they were not included in the model selection because of high correlation (Spearman) with other variables [e.g., CDD (INSECT) and CDD (PLANT)].

#### Morphometric data processing

The Euclidean distance from lakes or rivers and morphometric parameters (e.g., altitude) were processed in ArcGIS Pro and then extracted from the raster file for each monitoring record by using an automation model built with Model Builder function (Bajjali, 2023). Digital elevation models (DEMs) were obtained by TINITALY/01 (https://tinitaly.pi.ingv.it/), which is currently considered the most accurate DEM covering the whole Italian territory (Tarquini et al., 2007). TINITALY/01 is a DEM in triangular irregular network format created for the entire Italian territory in the UTM 32 WGS 84 coordinate system (Tarquini et al., 2007). The whole TINITALY/01 DEM was converted in grid format (10-m cell size) according to a tiled structure composed of 193.50-km side square elements (Mascandola et al., 2021). DEM coordinates were assigned as WGS 1984 UTM Zone 32N (WKID 32632).

### Statistical analysis

*Bactrocera oleae* infestation was evaluated as the occurrence of active infestation at 1% threshold and as the Julian day of occurrence of the first attacks. Each dependent variable was analyzed in two different periods, that is, July–August (i.e., early season) and September–October (i.e., late season) of each year from 2015 to 2021. The initial explanatory variables considered were DEM, LAKES, TEMP (MAR–MAY), TEMP (NOV–FEB), CDD (PLANT), TEMP (−7D), and SWB (**Table 1**). Considered orchards were monovarietal or mixed olive cultivars. To evaluate the effect of cultivars, we have tested the effect of a four-level categorical variable (cultivar, CV). Three levels were Leccino, Frantoio, and Moraiolo varieties. A fourth level grouped mostly olive plantations with mixed cultivars or with cultivars marginally represented in the dataset (e.g., “Nostrale di Rigali”). Agronomic management has a variable effect on *B. oleae* attacks (Gkisakis et al., 2018). A dummy variable (management, MAN) was included to compare the two management systems applied in olive orchards, i.e., IPM vs. organic. However, neither CV nor MAN variables revealed a significant effect on *B. oleae* occurrence of active infestation and Julian day of occurrence of first attacks; hence, they were not retained in the final models (see **Supplementary Tables 1**, **2** for statistical results). Similarly, the Euclidean distance from the rivers was initially evaluated, but its effect on *B. oleae* attacks was never significant. All variables were standardized (mean-centered with a unit standard deviation) prior to analysis. Attack probability was initially evaluated by means of generalized mixed-effects models (with logit link and binomial distribution) to account for dependent observations (multiple observations on the same orchard across different years) (**Supplementary Tables 1**, **2**). The Julian day of occurrence of the first attacks was evaluated by means of linear mixed-effects models (Burnham and Anderson, 2002; Rondoni et al., 2012), excluding from the final analysis the data from 2017, due to the limited number of infestation outbreaks. For both types of models, the relevance of the fixed and random structure was evaluated by means of the likelihood ratio test (LRT) and Akaike information criteria (AIC) (Pinheiro and Bates, 2006; Jacobs et al., 2022; Tidau et al., 2023). The best-fitted models, i.e., retaining the minimum number of explanatory variables, were selected using LRT (Burnham and Anderson, 2002; Ferracini et al., 2023). The multicollinearity of variables was assessed through the calculation of the variance inflation factor and revealed low (Zuur et al., 2009). A residual plot was evaluated for each of the best-fitted models. Data were analyzed and visualized using “MASS” (Venables and Ripley, 2002), “nlme” (Pinheiro and Bates, 2006), “lme4” (Bates et al., 2015), “ciTools” (Haman and Avery, 2020), “ggplot2” (Wickham, 2016), and “ggeffects” (Lüdecke, 2018) packages in R (version 4.2.2) (R Core Team, 2022).

## Results

**Figure 1:**
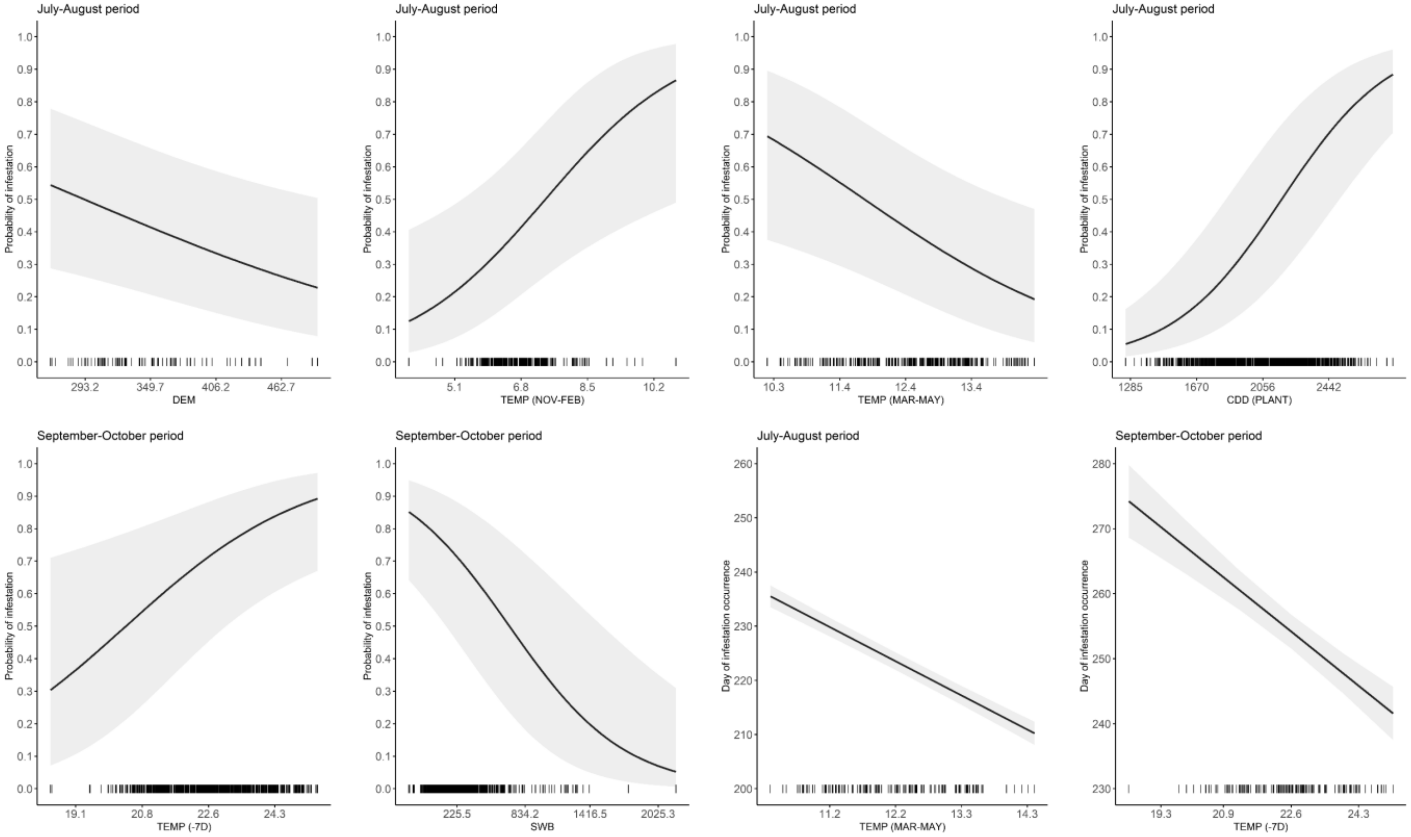
Relationships between infestation probability or Julian day of occurrence of *Bactrocera oleae* and some of the variables retained in the best-fitted models of **Tables 2**–**5**. Curves represent the model estimate (solid line) and 95% confidence intervals (shaded area). For each plot, the variables not represented were set to their average value on the original scale (reported in the caption of **Tables 2**–**5**). Tick symbols represent the distribution of the original data. Explanatory variables are described in **Table 1**.

The best model to explain the probability of attack in July–August retained five explanatory variables. Attack was negatively affected by DEM and TEMP (MAR–MAY), but positively affected by TEMP (NOV–FEB), CDD (PLANT), and SWB (the results of the best-fitted generalized mixed-effect model are reported in **Table 2**; **Figure 1**). Concerning the attacks in September–October, these were negatively affected by the increase of SWB and TEMP (MAR–MAY) but positively by TEMP (NOV–FEB) and TEMP (−7D) (**Table 3**; **Figure 1**). Concerning the day of the first attacks (**Table 4**), the increase in CDD (PLANT), DEM, and SWB delayed the occurrence of the attacks in the July–August period. On the contrary, an increase in LAKES, TEMP (NOV–FEB), TEMP (MAR–MAY), and TEMP (−7D) had a positive effect in anticipating the attacks. For the September–October period, higher values of DEM and CDD (PLANT) delayed the attacks, while an increase in TEMP (MAR–MAY) and TEMP (−7D) anticipated the attacks by *B. oleae* (**Table 5**).

**Table 2:** Coefficients and significance level for each explanatory variable retained within the best fitted model (Generalized linear mixed-effects model, binomial distribution) for the probability of occurrence of the first active infestation (1% threshold) within the period July-August of all years from 2015 to 2021.

Predictor	Coefficient	SE	z-value	P-value
(Intercept) DEM TEMP (NOV-FEB) TEMP (MAR-MAY) CDD (PLANT) SWB	-0.348 -0.342 0.558 -0.505 0.864 0.416	0.510 0.144 0.216 0.205 0.117 0.116	-0.68 -2.38 2.59 -2.46 7.35 3.59	0.495 0.017 0.010 0.014 <0.001 <0.001

Explanatory variables are described in **Table 1** and were standardized before analysis [average (SD) values: DEM = 349.7 (56.5) m, TEMP (NOV-FEB) = 6.8 (1.0) °C, TEMP (MAR-MAY) = 12.4 (0.9) °C, CDD (PLANT) = 2055.9 (275.4), SWB = 91.9 (221.3) mm].

Random effects: σ^2^ (variance of residuals) = 3.29, τ_00 st_ (variance between sites) = 0.66, τ_00 year_ (variance between years) = 1.67, ICC (intra-class correlation) = 0.41, N _st_ (number of sites) = 79, N _year_ (number of years) = 7, observations = 981, marginal R^2^ = 0.176, conditional R^2^ = 0.518, AIC = 971.97.

**Table 3:** Coefficients and significance level for each explanatory variable retained within the best fitted model (Generalized linear mixed-effects model, binomial distribution) for the probability of occurrence of the first active infestation (1% threshold) within the period September-October of all years from 2015 to 2021.

Predictor	Coefficient	SE	z-value	P-value
(Intercept) TEMP (NOV-FEB) TEMP (MAR-MAY) TEMP (-7D) SWB	0.91 0.570 -0.653 0.523 -0.514	0.566 0.270 0.313 0.201 0.125	1.61 2.11 -2.09 2.61 -4.13	0.108 0.035 0.037 0.009 <0.001

Explanatory variables are described in **Table 1** and were standardized before analysis [average (SD) values: TEMP (NOV-FEB) = 6.6 (1.0) °C, TEMP (MAR-MAY) = 12.5 (0.99) °C, TEMP (-7D) = 22.6 (1.2) °C, SWB = 225.5 (264.7) mm].

Random effects: σ^2^ = 3.29, τ_00 st_ = 0.52, τ_00 year_ = 1.94, ICC = 0.43, N _st_ = 75, N _year_ = 7, observations = 763, marginal R^2^ = 0.137, conditional R^2^ = 0.506, AIC = 798.18.

**Table 4:** Coefficients and significance level for each explanatory variable retained within the best fitted model (Linear mixed-effects model) for Julian day of the occurrence of the first active infestation (1% threshold) within the period July-August in the period 2015 to 2021.

Predictor	Coefficient	SE	z-value	P-value
(Intercept) DEM LAKES TEMP (NOV-FEB) TEMP (MAR-MAY) CDD (PLANT) TEMP (-7D) SWB	223.53 0.403 -0.59 -1.852 -5.733 15.835 -7.821 0.584	0.808 0.157 0.187 0.266 0.296 0.269 0.364 0.177	276.54 2.56 -3.16 -6.97 -19.36 58.92 -21.46 3.31	<0.001 0.012 0.002 <0.001 <0.001 <0.001 <0.001 0.001

Because of the low infestation events, 2017 was excluded from the analysis.

Explanatory variables are described in **Table 1** and were standardized before analysis [average (SD) values: DEM = 345.9 (54.0) m, LAKES = 8463.0 (5991.0) m, TEMP (NOV-FEB) = 7.0 (0.9) °C, TEMP (MAR-MAY) = 12.2 (1.0) °C, CDD (PLANT) = 2022.4 (261.7), TEMP (-7D) = 21.9 (1.9) °C, SWB = 152.5 (244.8) mm].

Random effects: σ^2^ = 2.19, τ_00 year_ = 3.77, N _year_ = 6, observations = 139, marginal R^2^ = 0.932, conditional R^2^ = 0.975, AIC = 544.76.

**Table 5:** Coefficients and significance level for each explanatory variable retained within the best fitted model (Linear mixed-effects model) for Julian day of the occurrence of the first active infestation (1% threshold) within the period September-October in the period 2015 to 2021.

Predictor	Coefficient	SE	z-value	P-value
(Intercept) DEM TEMP (MAR-MAY) CDD (PLANT) TEMP (-7D)	254.17 1.437 -2.64 7.374 -6.425	1.311 0.527 1.055 0.884 0.803	193.84 2.72 -2.5 8.35 -8.00	<0.001 0.007 0.014 <0.001 <0.001

Because of the low infestation events, 2017 was excluded from the analysis.

Explanatory variables are described in **Table 1** and were standardized before analysis [average (SD) values: DEM = 347.4 (52.6) m, TEMP (MAR-MAY) = 12.4 (1.0) °C, CDD (PLANT) = 2624.8 (295.5), TEMP (-7D) = 22.6 (1.3) °C].

Random effects: σ^2^ = 34.94, τ_00 year_ = 8.4, N _year_ = 6, observations = 135, marginal R^2^ = 0.541, conditional R^2^ = 0.630, AIC = 875.55.

## Discussion

This study represents the first attempt to understand *B. oleae* population dynamics in Umbria (Central Italy) using a combination of landscape and agroclimatic variables, which could be further leveraged in studies related to larger territories with similar geographical characteristics. The assessment of *B. oleae* infestation was carried out over two crucial periods of the year, that is, in July–August, during the fruit growth period, and in September–October, during the preharvest period.

Our analysis reveals that olive orchards located at higher altitude expected lower attacks during summer (July–August period). In these sites, attacks are also delayed during the year (July–August and September–October periods). Similarly, Helvaci et al. (2018) reported that the infestation rate was inversely related to both altitude and relative humidity in olive orchards of Northern Cyprus. Furthermore, temperatures in winter and spring have a strong effect on determining attacks and timing of infestation. Average daily temperatures were approximately 7°C in November–February, i.e., when *B. oleae* is mostly overwintering, and determined an attack probability of approximately 50% (averaged across years). Temperature increase in this period has a positive effect in enhancing future attacks throughout the year, e.g., with an increase in the early season of ca. 35% probability of attacks at 8.5°C average temperatures. Our analysis suggests that temperatures also anticipate the timing of attacks during the early season period of approximately 2 d per degree. Insects that overwinter as pupa in soil have increased survival when temperature increases above 1°C (Bale and Hayward, 2010). Winter temperatures above 0°C gradually reduce the mortality of *B. oleae*, which can successfully overwinter with large populations (Hatherly et al., 2005; Wang et al., 2013; Petacchi et al., 2015; Marchi et al., 2016). Similarly, Marchi et al. (2016) registered an anticipation of *B. oleae* appearance in spring and higher infestation rates of juvenile forms (i.e., eggs, first and second instar larvae alive and dead) during the early season period (July to August) in years characterized by mild winters. Helvaci et al. (2018) detected a higher infestation rate as a consequence of higher winter air temperatures.

The increase of the average temperatures in the March–May period negatively affects attack probability but remarkably anticipates the occurrence of first attacks. Given the massive influence of temperature on insects and the fact that higher temperatures often lead to shorter life spans, the combination of a mild winter and a hot spring could potentially be responsible for the premature decline of overwintering adults (Preu et al., 2020), possibly resulting in a lower attack probability. In non-irrigated orchards, such as those investigated here, warmer spring temperatures may also negatively affect pupal survival, whereas irrigation may prevent pupal desiccation (reviewed by Yokoyama, 2015). In addition, during spring, most of the newly emerged *B. oleae* adults disperse from olive orchards, where fruits are not available, to seek flowers and nectar for survival (Paredes et al., 2023). During these migratory flights, newly hatched adults can also encounter abandoned olive groves, where they can find fruits from the previous year to oviposit. In this scenario, the reduced infestation found in the surveyed olive groves might be due to the higher spring temperatures followed by an increased frequency and distance of migratory flights (Economopoulos et al., 1978; Mazomenos et al., 2002; Ragaglini et al., 2007; Skouras et al., 2007; Marchini et al., 2017; Ortega et al., 2022). The part of the population that did not migrate, on the other hand, may have been responsible for the early attacks recorded.

More hypotheses could be drawn for the observed negative effect of increased spring temperatures on attacks and could, for example, consider the modification of the chemical and physical factors involved in the susceptibility of olives to *B. oleae* (Tognetti et al., 2006; Malheiro et al., 2015).

The accumulation of degree days relevant to the olive phenology positively determined *B. oleae* attacks but only for the July–August period, confirming the results of previous studies conducted in other Italian regions (Marchi et al., 2016; Volpi et al., 2020).

Notably, higher temperatures in the 7-day period before the monitoring day increase the attack probability only in the late season and anticipate the occurrence of attacks in both early and late seasons. Other variables rather than temperature may affect the interactions between olive fruit fly and its host, such as soil water content and irrigation type. For example, we detected that an increase in the soil water content increases the attack probability by *B. oleae.* A low water level can lead to loss of plant turgor and premature fruit drop. Conversely, adequate water supply improves fruit turgidity, which elicits higher *B. oleae* attacks on fruits (Marchi et al., 2016). Irrigation practice was not considered within the model selection because in Central Italy productive olive plantations are not irrigated. Vicinity to lakes is known to increase the attack probability by *B. oleae* because of the peculiar microclimate caused by lakes (Gutierrez et al., 2009). However, in our analysis, proximity to lakes was of marginal importance and never affected the probability of attacks. An explanation is that a possible variability in the attacks in orchards gradually distant from lakes could have been captured by other variables considered (e.g., winter and spring temperatures).

Collectively, our results support a potential application of IPM control strategies leveraging predictive models in the future implementation of decision support systems. The main bottleneck of this application is the evaluation of false negatives that can underestimate the infestation rates in olive orchards (Volpi et al., 2020). For these reasons, different interpolation methods must be tested before choosing the best-fit model for a specific geographic and climatic area (Petacchi et al., 2015). In the case of *B. oleae*, adequate monitoring networks are needed to implement a proper program, as insect distribution is affected by environmental and landscape factors (Thies et al., 2003; Krasnov et al., 2019). Despite the difficulties of precision agriculture strategy for pest control, there are previous studies confirming the successful implementation of digital tools (Miranda et al., 2019). Sciarretta et al. (2019) reported a substantial reduction in the number and volume of pesticide applications, for the control of the Mediterranean fruit fly (medfly), *Ceratitis capitata* (Wiedermann), in areas managed with a dedicated decision support system and electronic monitoring traps. Given the close relationship between insect development and temperature, further studies on the impact of temperature variations on the spatial and temporal distribution of *B. oleae* populations are urgently needed. In addition to air temperature, the emergence of *B. oleae* can be affected by soil temperature and moisture. For example, low soil temperature and high soil moisture due to rain can increase *B. oleae* pupal mortality (Neuenschwander et al., 1981; Wang et al., 2013). Conversely, low soil humidity may be responsible for pupal desiccation, drastically reducing adult emergence in the spring (Wang et al., 2013; Yokoyama, 2015). The use of specific sensors to measure soil parameters would be necessary to further elucidate the effect of soil temperature and moisture on the overwintering population of *B. oleae*.

Moreover, traditional control methods (i.e., cover and bait sprays) based on conventional insecticides could become inefficient if the olive fruit fly continues to increase its resistance (Skouras et al., 2007; Pereira-Castro et al., 2015; Marchi et al., 2016). Alternative approaches, by combining powder dust or kaolin with propolis, exhibited an interesting and concrete possibility to reduce fruit infestations by *B. oleae* (Daher et al., 2022). An integration of different olive fruit fly management methods is recommended, including the use of chemical, biotechnical, and biological control (Lantero et al., 2023). The complexity of the landscape surrounding olive groves has been shown to reduce the abundance of *B. oleae* and other insect pests (Ortega et al., 2018; Villa et al., 2020). In addition, a more complex environment has been found to enhance the effectiveness of biocontrol agents (Boccaccio and Petacchi, 2009; Ortega et al., 2018). Therefore, agricultural spatial planning should consider the impact of landscape complexity on insect development. Our analysis allowed the identification of key environmental variables influencing the probability and timing of *B. oleae* attacks that could be used in distribution modeling at the regional scale, or even in more complex modeling methods, based on machine learning algorithms. Further analyses should also evaluate the effect of landscape complexity and composition on olive fruit fly infestations occurring in Central Italy.

## Data availability statement

Data will be provided upon request directed to GR (gabriele.rondoni@unipg.it).

## Ethics statement

The manuscript presents research on animals that do not require ethical approval for their study.

## Author contributions

GR: Conceptualization, Formal analysis, Methodology, Writing – original draft, Writing – review & editing, Supervision. EM: Methodology, Writing – original draft, Writing – review & editing, Conceptualization, Formal analysis. VAG: Methodology, Writing – original draft, Writing – review & editing. ElC: Methodology, Writing – review & editing, Writing – original draft. AB: Conceptualization, Investigation, Methodology, Writing – review & editing, Supervision. GN: Writing – review & editing, Conceptualization, Investigation, Methodology. RP: Writing – review & editing. FF: Conceptualization, Supervision, Writing – review & editing. AN: Conceptualization, Methodology, Supervision, Writing – review & editing. ErC: Conceptualization, Methodology, Supervision, Writing – review & editing.
