# Supplementary material for: Evaluation of the effect of agroclimatic variables on the probability and timing of olive fruit fly attack
Source: Front Plant Sci. 2024 Jul 15;15:1401669. doi: 10.3389/fpls.2024.1401669 (PMC11284310; doi:10.3389/fpls.2024.1401669)
Supplement: Supplementary file 1 [file DataSheet_1.pdf]

Table S1: Comparisons of the different models for the effect of explanatory variables on the probability of occurrence of the first active infestation (1% threshold) within the period July-August or September-October of all years from 2015 to 2021. The best fit model is highlighted in bold.

Response variable	Explanatory variables	random intercept	AIC	Model comparison
Months: July-August	model #1: DEM, LAKES, TEMP (MAR-MAY), TEMP (NOV-FEB), CDD (PLANT), TEMP (-7D), SWB, MAN, CV	YEAR, ORCHARD	981.17	vs. best model: ΔAIC = 9.21, Chisq = 2.79, P = 0.83
	**model #2: DEM, TEMP (MAR-MAY), TEMP (NOV-FEB), CDD (PLANT), SWB**	**YEAR, ORCHARD**	**971.97**	**vs. intercept only model: ΔAIC = 114.04, Chisq = 124.04, P < 0.0001**
Months: September-October	model #1: DEM, LAKES, TEMP (MAR-MAY), TEMP (NOV-FEB), CDD (PLANT), TEMP (-7D), SWB, MAN, CV	YEAR, ORCHARD	806.08	vs. best model: ΔAIC = 7.90, Chisq = 6.10, P = 0.53
	**model #2: TEMP (MAR-MAY), TEMP (NOV-FEB), TEMP (-7D), SWB**	**YEAR, ORCHARD**	**798.18**	**vs. intercept only model: ΔAIC = 25.02, Chisq = 33.02, P < 0.0001**

Table S2: Comparisons of the different models for the effect of explanatory variables on the Julian day of the occurrence of the first active infestation within the period July-August or September-October (years 2015, 2016, 2018, 2019, 2020, 2021). The best fit model is highlighted in bold.

Response variable	Explanatory variables	random intercept	AIC	Model comparison
Months: July-August	model #1: DEM, LAKES, TEMP (MAR-MAY), TEMP (NOV-FEB), CDD (PLANT), TEMP (-7D), SWB, MAN, CV	YEAR	547.44	vs. best model: ΔAIC = 2.68, L. Ratio = 6.22, P = 0.18
	**model #2: DEM, LAKES, TEMP (MAR-MAY), TEMP (NOV-FEB), CDD (PLANT), TEMP (-7D), SWB**	**YEAR**	**544.76**	**vs. intercept only model: ΔAIC = 446.42, L. Ratio = 472.56, P < 0.0001**
Months: September-October	model #1: DEM, LAKES, TEMP (MAR-MAY), TEMP (NOV-FEB), CDD (PLANT), TEMP (-7D), SWB, MAN, CV	YEAR	868.7	vs. best model: ΔAIC = -6.85, L. Ratio = 6.41, P = 0.49
	**model #2: TEMP (MAR-MAY), CDD (PLANT), TEMP (-7D), DEM**	**YEAR**	**875.55**	**vs. intercept only model: ΔAIC = 83.09, L. Ratio = 87.44, P < 0.0001**
